# Optimizing power consumption and position control in an electro-hydraulic system with cylinder bypass and NN-MPC

**DOI:** 10.1038/s41598-024-51318-x

**Published:** 2024-01-23

**Authors:** Omar Hesham Khedr, Mohammed Ibrahim Awad, Affaf Khamis Al-Oufy, Shady A. Maged

**Affiliations:** 1 Mechatronics Engineering Department, Faculty of Engineering, Ain Shams University, Cairo, Egypt; 2 Mechanical Engineering Department, Faculty of Engineering, Galala University, Suez, Egypt; 3 Textile Engineering Department, Faculty of Engineering, Alexandria University, Alexandria, Egypt

**Keywords:** Engineering, Mechanical engineering

## Abstract

This study introduces an innovative approach to enhance the energy efficiency and position control performance of electro-hydraulic systems, employing a comprehensive comparative analysis. It presents and evaluates three control techniques: Proportional-Integral-Derivative (PID) control, Model Predictive Control (MPC), and Neural Network Model Predictive Control (NN-MPC). These methods are systematically assessed across varying load conditions. Notably, our research unequivocally establishes the exceptional performance of the NN-MPC approach, even when confronted with load variations. Furthermore, the study conducts an exhaustive examination of energy consumption by comparing a conventional system, where a flow control valve is not utilized as a hydraulic cylinder bypass, with a proposed system that employs a fully open Flow Control Valve (FCV). The results underscore the remarkable energy savings achieved, reaching up to 9% at high load levels.

## Introduction

Electrohydraulic systems (EHS) have garnered significant attention in diverse industrial settings for their remarkable performance in power generation and positioning. These systems possess outstanding versatility and are widely used in sectors such as construction, modern airspace, mining, and manufacturing^1^. One of the key factors that contribute to their popularity is their impressive power-to-weight ratio, rapid response, exceptional positioning capabilities, and high durability, as pointed out by^2^.

Despite the many advantages of Electrohydraulic systems in various applications related to positioning and power generation, they suffer from several limitations, including throttling, overflowing, low efficiency, complexity, nonlinearity, uncertainty, and external disturbances. Traditional control techniques have difficulty in addressing these challenges, making it necessary to explore alternative solutions. This study aims to use machine learning algorithms and energy-efficient position control techniques to overcome these issues, utilizing an innovative circuit that incorporates conventional hydraulic components. The following paragraphs will delve into the latest and most significant research studies on EHS, providing a comprehensive summary of the current state of the art in EHS research and development.

Numerous control techniques, such as the traditional PID, LQR, and others, have been thoroughly examined and verified by researchers^3–5^. Despite their widespread usage, these standard control theories frequently fall short in accurately tracking the desired reference due to the inherent uncertainties and nonlinearities of EHS. Subsequently, researchers have delved into exploring nonlinear controllers to enhance the actuator position’s control accuracy.

To overcome the nonlinearities of EHS, a nonlinear controller combining sliding mode control and back-stepping technique was suggested by^6^. Meanwhile, a standard sliding mode control (SMC) was developed by^7^ to overcome the uncertainties and nonlinearities brought on by friction and internal leakages, and its performance was compared with that of a PID controller. Additionally, various inputs in the form of steps and sinusoids with different frequencies were applied to the system to track the SMC’s performance.

In order to reduce the effects of delay on control performance, a predictive control approach was proposed by^8^, which was utilized to control EHS and achieved superior control performance compared to other techniques. Furthermore^9^, developed a simulation model of sliding mode control implemented in EHS and compared the results with those of a robust controller and Model Predictive Control. Finally, to improve the robustness and dynamic properties of EHS^10^, employed a hybrid controller that combines MPC and PI controller, as MPC has been scarcely studied in the context of EHS.

In contemporary times, the conservation of power has become an increasingly pressing issue, prompting researchers to explore innovative solutions. One study^11^ investigates the use of electro-hydraulic systems and suggests that the energy consumption of EHS can be reduced by concurrently controlling the actuator position and supply pressure. To achieve this, the pressure relief valve’s setting is adjusted according to the spool position of the proportional directional valve. The study employs simulation and testing to demonstrate that this approach leads to the optimal reduction of EHS’s overall power consumption. The researchers also suggest using fixed displacement pumps as an additional measure to further lower energy consumption in electro-hydraulic systems.

Furthermore, a ground-breaking investigation by^12^ proposes a unique hydrostatic system that uses a flywheel as an energy storage device, significantly lowering energy usage in compared to conventional hydrostatic motors. Test findings show that the recently proposed system only makes use of 52% of the energy from the main power source. The efficiency of the system is especially useful for mobile hydraulic machinery applications.

To improve the energy efficiency of hydraulic elevators^13^, suggest using a hydraulic system based on a Variable Voltage Variable Frequency (VVVF) controller. This system uses the accumulator pressure as an energy storage and release unit, reducing the amount of installed energy and overall energy consumption. The study by^14^ delves into the optimization of linear actuator performance, which holds paramount importance in heavy earth moving equipment utilized across construction and mining sectors. Their comparative analysis of two hydraulic circuits, employing a conventional proportional directional control valve (PDCV) and an inventive integration of a proportional flow control valve (PFCV) to regulate artificial leakage, highlights the pursuit of enhanced energy efficiency and precise control.

Furthermore, within the realm of using FCV^15^, investigates the static and dynamic characteristics of a proportional flow control valve designed explicitly for use in excavators. The paper meticulously examines the performance-related parameters of this valve, shedding light on its suitability for achieving both energy savings and optimal control in excavator applications. This exploration into the proportional flow control valve’s attributes aligns with the ongoing pursuit of enhancing energy efficiency and precision in heavy machinery

Lastly, reference^16^ introduces the leaking valve pump parallel control (LVPC) system, which is designed to enhance the response of the variable speed pump control (VSPC) system. The LVPC system achieves this by incorporating a leaking control valve in parallel with the variable speed pump, thereby regulating the system flow and resulting in a reduction of energy consumption.

This paper proposes a novel approach to achieve two primary goals in hydraulic systems: power conservation and position tracking. Previous studies^8–10^ have shown that MPC controllers can improve dynamic response in systems with nonlinearities, but traditional controllers have limitations such as time delays, overshooting, chattering, and tracking errors. To address these issues, this paper employs a neural network MPC controller, which effectively reduces nonlinearities and improves control performance.

In addition, a comparison is made between two hydraulic circuits: a conventional proportional directional control valve (PDCV) and a flow control valve (FCV) that employs a hydraulic cylinder bypass. Experimental findings demonstrate that the FCV circuit outperforms the PDCV circuit, resulting in significant reductions in energy consumption (by 15.35% under no-load conditions and 9% at 7.5 MPa) and average operating temperature (by 10%). The proposed approach is further validated using simulation and experimental results, thus confirming its practicality and effectiveness.

The present study aims to provide a comprehensive understanding of the mathematical modeling and experimental setup of a hydraulic system. To this end, the paper is divided into several sections, each covering a specific aspect of the research. In Section "Developing a linear discrete-time state-space model", the hydraulic circuits and their components are explained in detail. Moving on, Section "Controllers design and simulation" presents the inputs and outputs collected through LabVIEW and NI-USB 6009. The state space of the system is then demonstrated using MATLAB’s Linear Analysis toolbox. In Section "PID controller design", the structure of the MPC, NN MPC, and PID controllers is described, along with the system simulation. The effectiveness of these controllers is compared in Section "The experiment and result", where experimental results under various loads are presented and contrasted with those from other EHS position control theories. In Section "Conclusion", the power consumption of the proposed circuits is compared, and their average operating temperature is evaluated. The paper concludes with a summary of the main findings and contributions of this research.

### System description and mathematical modeling of electro-hydraulic system

**Figure 1:**
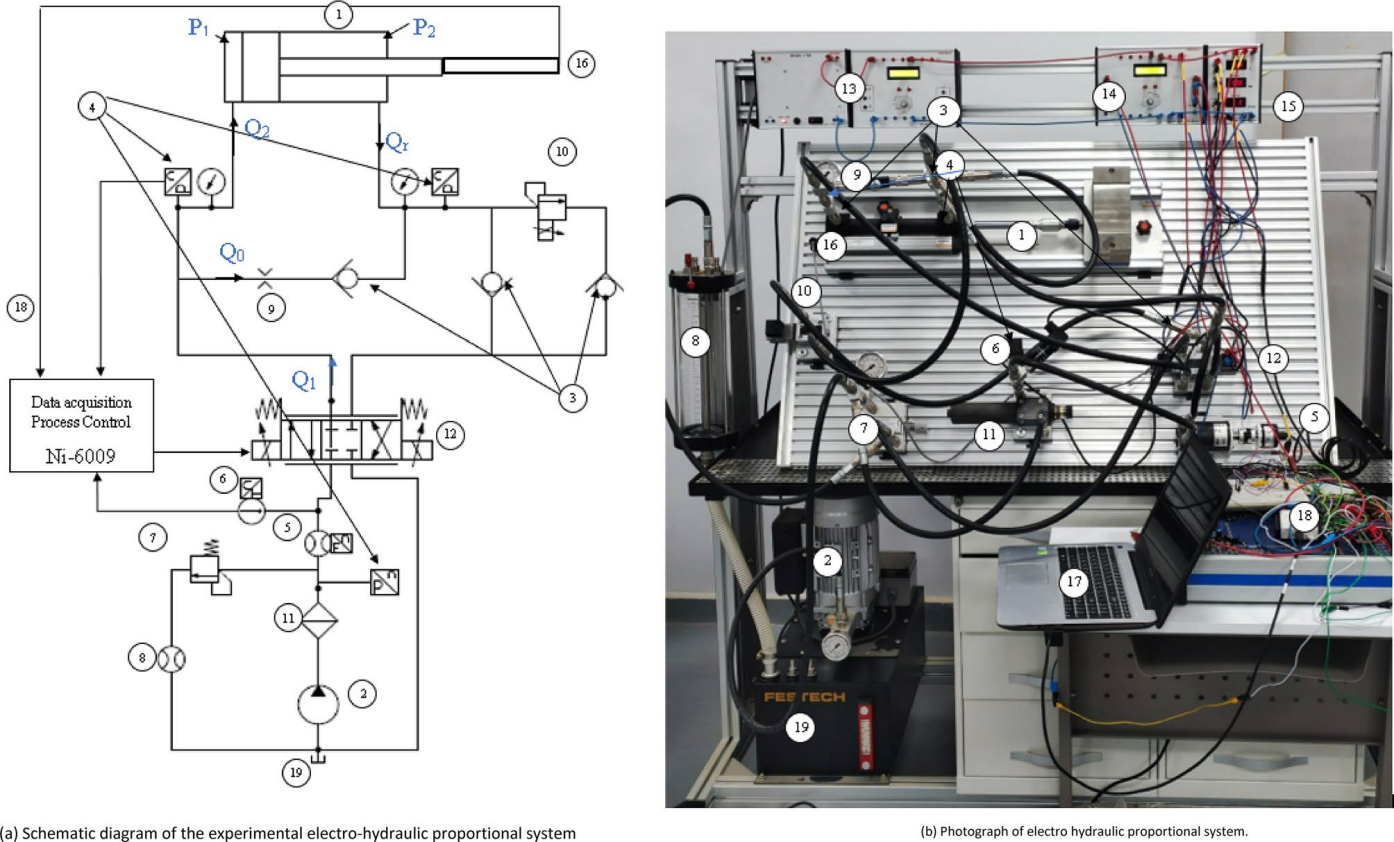
(**a**) Schematic diagram of the experimental electro-hydraulic proportional system. (**b**) Photograph of electro hydraulic proportional system.

Figure 1 presents our laboratory’s experimental setup. The schematic depiction in Fig. 1a details the interconnections among system components, elucidating their roles in enabling precise control of the hydraulic cylinder’s position. This diagram visually maps the flow pathway of hydraulic fluid, showcasing the positioning and relationships of crucial elements: the hydraulic cylinder (1), gear pump (2), valves [including the proportional directional control valve (12) and relief valve (7)], pressure transducers (4), flow meter (5), filters (11), measuring units (8), amplifier circuit (14), linear potentiometer (16), and computer (17) utilized for data acquisition and analysis. Moreover, Fig. 1b offers a tangible representation through a photograph, providing a realistic view of the physical arrangement of the electro-hydraulic system. This image substantiates the assembly of components delineated in the schematic diagram. Additionally, a summarized overview of the system components is presented in Table 1, specifying the description and roles of each constituent element.

**Table 1:** Components of the experimental hydraulic system.

Component	Description
Hydraulic cylinder (1)	Contains a 25 mm piston diameter
Gear pump (2)	A pump powered by a 1.5 KW AC motor to supply hydraulic fluid to the system
Ball check valve (3)	Prevents reverse flow and ensures one-way fluid movement
Pressure transducers and gauges (4)	Instruments to measure pressure at the rod and bore ends of the cylinder, as well as pump outlet pressure
Flowmeter (5)	Measures and records the flowrate delivered to the hydraulic cylinder
Hydraulic tank (6)	Stores hydraulic fluid and acts as a reservoir
Main pressure relief valve (PRV) (7)	Safeguard to bypass excess flow and maintain system pressure
Measuring unit (8)	Records various parameters such as pressure, flow
FCV (9)	A flow control valve utilized as a bypass between the inlet and outlet of the cylinder, providing control over the flow of hydraulic fluid within the system
Proportional relief valve (10)	Used for simulating loads by restricting the oil flow exiting the cylinder with hydraulic pressure that is proportional to the signal provided to the Proportional Amplifier, ranging from 0 to 10 V
Pressure filter (11)	Removes impurities and contaminants from the hydraulic fluid
4/3 PDCV (12)	A 4/3 proportional directional control valve used to regulate flow during cylinder position control
Amplifier circuit (14)	Amplifies the signal output from data acquisition, converting it from 0 to 5 V to the required input range of the Proportional Amplifier for the PDCV, which operates from − 10 to 10 V
Linear potentiometer (16)	Provides feedback on the cylinder’s position for control and monitoring purposes
Computer (17)	Equipped with a data acquisition card for recording and analyzing the cylinder’s response

The development of a controller requires a thorough mathematical modelling of the system. In this study, the dynamics of an electrohydraulic proportional system are mathematically modelled, under certain assumptions. Specifically, fluid leakage in the cylinder and valve, as well as contact between the cylinder and piston, are ignored. The mathematical models for the two hydraulic systems are presented below, with Eqs. (1) to (9) providing an explanation for each. First, the system equations for the hydraulic circuit using only PDCV are expressed^17^.

1 $${Q}_{L}=\frac{{Q}_{2}+{Q}_{r}}{2}$$

Equation (1) characterizes the flow of the load inside the cylinder, where ${Q}_{2}$ denotes the inlet flow and ${Q}_{r}$ denotes the exit flow.

2 $${\text{P}}_{{\text{L}}} = {\text{P}}_{1} - {\text{P}}_{2}$$

Equation (2) represents the pressure of the load on the cylinder, which can be calculated by finding the difference between the inlet and exit pressures of the cylinder.

3 $${Q}_{2}=\left[{\frac{V}{\beta }\dot{P}}_{1}+A Vp\right]$$

Equation (3) represents the flow that propels the actuator’s movement and corresponds to the compressible flow present in the cylinder chamber.

4 $${Q}_{r}=\left[-{\frac{V}{\beta }\dot{P}}_{2}+A Vp\right]$$

Equation (4) represents the flow leaving the cylinder, which is obtained by subtracting the compressible fluid in the cylinder from the flow generated by the actuator motion.

5 $${Q}_{L}=\left[{\frac{V}{2\upbeta }\dot{P}}_{L}+A Vp\right]={K}_{f} Xv-{K}_{t} {P}_{L}$$

By incorporating the information from the four equations stated previously, Eq. (5) characterizes the load flow equation inside the cylinder and through the proportional valve. This equation is also dependent on the spool position and the load pressure.

6 $$F=A {P}_{L} =m\dot{V}p+b Vp+{F}_{L}$$

In Eq. (6), the balance of pressure force is illustrated, taking into account the external load acting on the cylinder, the viscous friction, and the inertial force.

The system equations of the hydraulic system using FCV are stated down below:

7 $${Q}_{1}={Q}_{0}+{Q}_{2}$$

Equation (7) illustrates that the flow generated by the pump is equal to the summation of the flow through the flow control valve and the flow entering the cylinder. Moreover, Eq. (8) showcases the mathematical representation for the flow rate (Q_0_) of fluid passing through an orifice.

8 $$Q_{0 = } C_{d} A\sqrt {\frac{{2P_{1} }}{\rho } = K_{f} Xv - K_{t} P_{1} }$$

To provide a more comprehensive understanding of this principle, Eq. (9) offers a detailed expression for the flow Q_1_. It can be expressed as:

9 $${Q}_{1}={\frac{V}{2\upbeta }\dot{P}}_{1}+A Vp+ {K}_{f} Xv-{K}_{t} {P}_{1}$$

Table 2 provides a list of symbols and their corresponding meanings, which are utilized in the aforementioned equations^18^.

**Table 2:** Mathematical model symbols.

Notation	Nomenclature	Value
$A$	The surface area of the hydraulic piston cylinder	633 cm^2^
${P}_{L}$	Hydraulic pressure between the inlet and outlet of the actuator piston	–
B	Damping coefficient	1000 Nm^−1^ s
${F}_{L}$	External load disturbance	–
$Xv$	Spool valve displacement	–
$\beta$	The bulk modulus of hydraulic oil	689*106 Pa
$Vp$	Piston’s velocity	–
$v$	The volume of oil in the cylinder	468/1003 m^3^
${K}_{f}$	Proportional valve coefficient	1.02 m^2^/sec
${K}_{t}$	Leakage flow coefficient	0 m^3^/Pa s
$m$	Mass at load	12 kg
${C}_{d}$	Valve discharge flow co-efficient	–
$Xv$	Valve spool position gain	0.0406*10–2 m/V
$\rho$	Density of oil	833.3 kg/m^3^

## Developing a linear discrete-time state-space model

**Figure 2:**
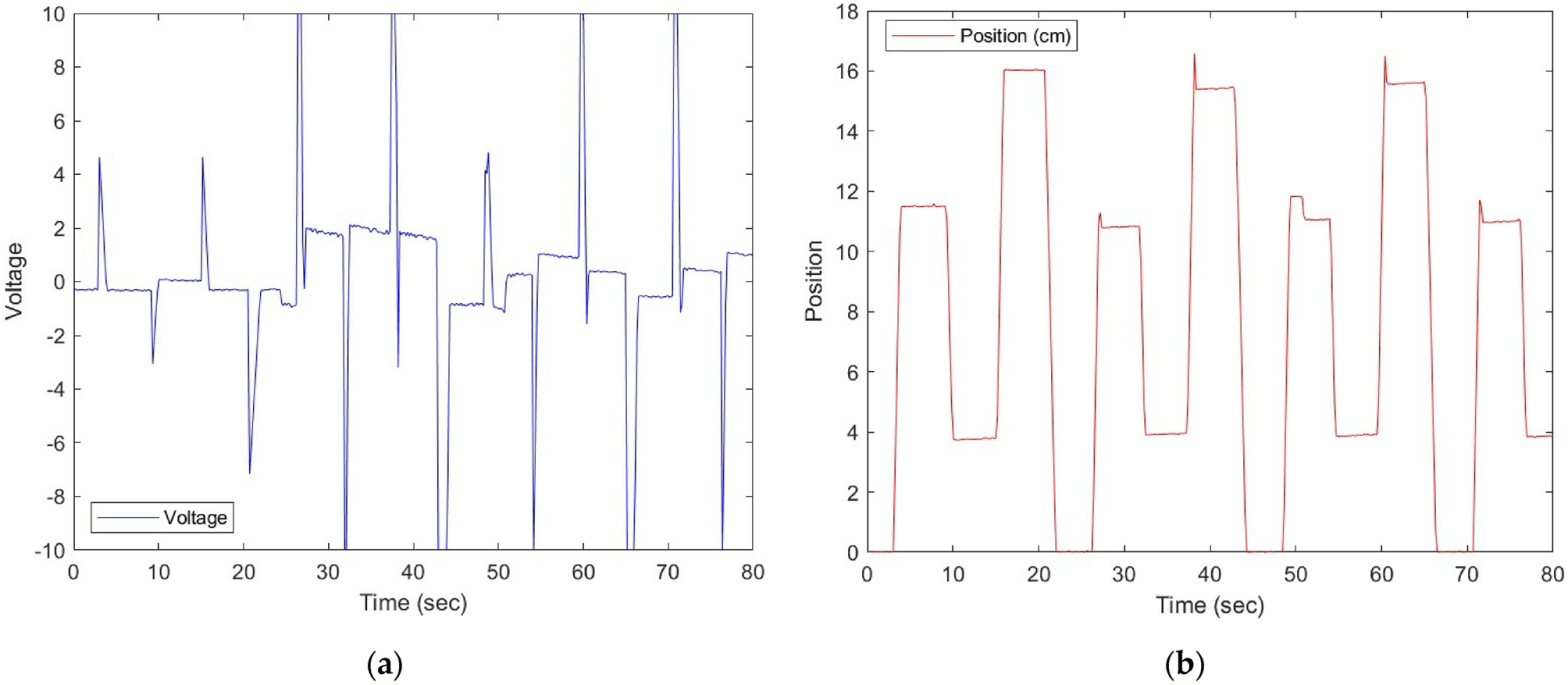
(**a**) Test signal for linear analysis tool. (**b**) Real-time response.

To implement Model Predictive Control in LabVIEW, a linear discrete-time state-space mathematical model must be established. This model is derived using MATLAB’s Linear Analysis Tool, and Eqs. (10) to (15) provide a detailed account of the process. To get the system’s time-domain response, random signals are used as input excitation. Figure 2 shows the input signal sent to the proportional amplifier of the PDCV and response of the closed-loop system^10^.

10 $$x(k+1)=A x(k) = B u(k)$$

11 $$Y(k)=C x(k)+D u(k)$$

12 $$A=\left[\begin{array}{c}\begin{array}{c}\begin{array}{ccc}1.018& -0.01259& 0.07238\\ -0.07183& 0.5918& 0.9991\\ -0.2108& -0.534& 0.8973\end{array}\end{array}\end{array}\right]$$

13 $$B=\left[\begin{array}{c}-0.002289\\ 0.0591\\ 0.0005919\end{array}\right]$$

14 $$C=\left[\begin{array}{ccc}71.64& -3.625& 0.3697\end{array}\right]$$

15 $$D= \left[\begin{array}{c}0\end{array}\right]$$

To evaluate the effectiveness of the control method, a simulation model is developed using the Control Design and Simulation Module in LabVIEW. The simulation allows for testing of the control method in a controlled environment, enabling an assessment of its efficacy before implementing it in real-world applications. The simulation results are then analyzed to determine if the controlled position accurately tracks the desired set-point.

## Controllers design and simulation

### Model predictive control

Model predictive control is a well-established controller that has found numerous successful applications in both academic and industrial settings. Its effectiveness and practicality have contributed to its widespread use. Moreover, MPC is capable of managing both measured and unmeasured disturbances. In order to improve the reference signal and estimate the output, measured noise (such as electrical noise and sampling errors) must be taken into consideration. MPC also offers feedforward compensation for both recorded and unmeasured disturbances^19–21^.

In MPC, a single discrete-time, linearly time-invariant, state-space system model is typically used to predict the system’s future behavior. The prediction horizon (P) and control horizon (M) are key components of the MPC definition. The value of M specifies the number of samples that will influence voltage, while P specifies the number of samples needed to estimate plant output. It is essential for M to have a lower value than P. The objective function J(k) is a quadratic nonlinear function. The equations from (17–19) specify its limitations on the outputs, control signal change, and control signal. The scalar system’s representation of J(k) is given by Eq. (16).

In the first step of the MPC technique, a single discrete-time, linearly time-invariant, state-space system model is typically used to predict the system’s future behavior. over a specified time horizon. At each instant ‘k’, predictions are made for the outputs ŷ(k+i), where ‘i’ ranges from 1 to ‘P’ for the prediction horizon. These predictions take into account previous inputs, outputs, and the future control signals u(k+i), where ‘i’ ranges from 1 to ‘M-1’.

In the second step, the MPC methodology computes a sequence of forthcoming control signals with the intention of optimizing an objective function and enhancing system performance. This objective function is denoted as ‘J(k)’ in Eq. (16), wherein ŷ stands for the output predicted by the model, R represents the set point, and ∆u indicates the change in manipulated input between successive sample instants. Additionally, r and q are used to symbolize the weights associated with these changes in input and errors in the predicted outputs, respectively. The variable ‘k’ denotes the present sample time.

The objective function is defined as:

16 $$J\left( k \right) = \mathop \sum \limits_{i = 1}^{P} q.\left[ { \hat{y}\left( {k + i} \right) - R\left( {k + i} \right)} \right]^{2} + \mathop \sum \limits_{i = 1}^{M - 1} r.\left[ {\Delta u\left( {k + i} \right)} \right]^{2}$$

Constraints on control signals, their rate of change, and the outputs are also incorporated into the cost function, as defined in Eqs. (17, 18, and 19).

17 $${u}_{min}\le u\left(k\right)\le {u}_{max}$$

18 $$\Delta ({u}_{min}) \le \Delta u(k) \le \Delta ({u}_{max})$$

19 $${y}_{min}\le y(k)\le {y}_{max}$$

In the third step of the MPC technique, the current control signal is applied to the system. In the subsequent interval, Step 1 is repeated while measuring the output using the receding horizon technique. This measurement is used to calculate the control signal for the next time step. Thus, the prediction horizon shifts into the future while maintaining the same length at each interval.

The choice of the control horizon is crucial, as a larger value increases the computational load, potentially leading to undesired performance. The output weighting and Equal Concern for Relaxation (ECR) weighting are significant considerations in tuning the MPC controller. In this study, the MPC controller is adjusted with a default control horizon value of 2 and a fixed output weighting value of 1. ECR weighting plays a role in balancing constraints against other performance goals, typically in the range of 5 to 20.

**Figure 3:**
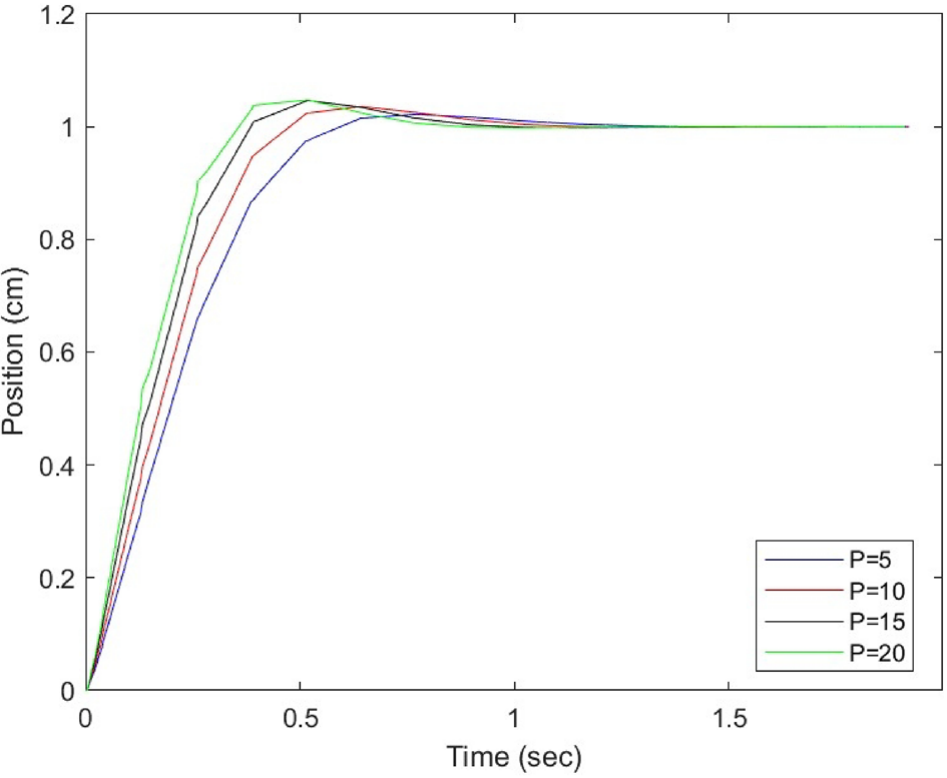
Simulation results of the MPC controller by tuning prediction horizons.

Figure 3 illustrates the output behavior of the MPC controller and demonstrates the impact of tuning the prediction horizons. The system’s performance is evaluated using unit step inputs to assess tracking control results. The results indicate that a prediction horizon of 15 is optimal in terms of tracking and control inputs.

In Table 3, the implemented parameters and cost weights for the model predictive controller are presented. The prediction horizon is set to 15, and the control horizon is set to 2. The MPC cost weights include the control action weight, control action rate weight, output weightings, and ECR weightings, each with their respective values:

**Table 3:** MPC controller parameters.

MPC controller parameters	
Prediction horizon	15
Control horizon	2
MPC cost weights	
Control action weight	0
Control action rate weight	0.1
Output weightings	1
ECR weightings	1

With this configuration, the LabVIEW MPC system is equipped with the necessary parameters and weights to achieve optimal control and tracking performance.

**Figure 4:**
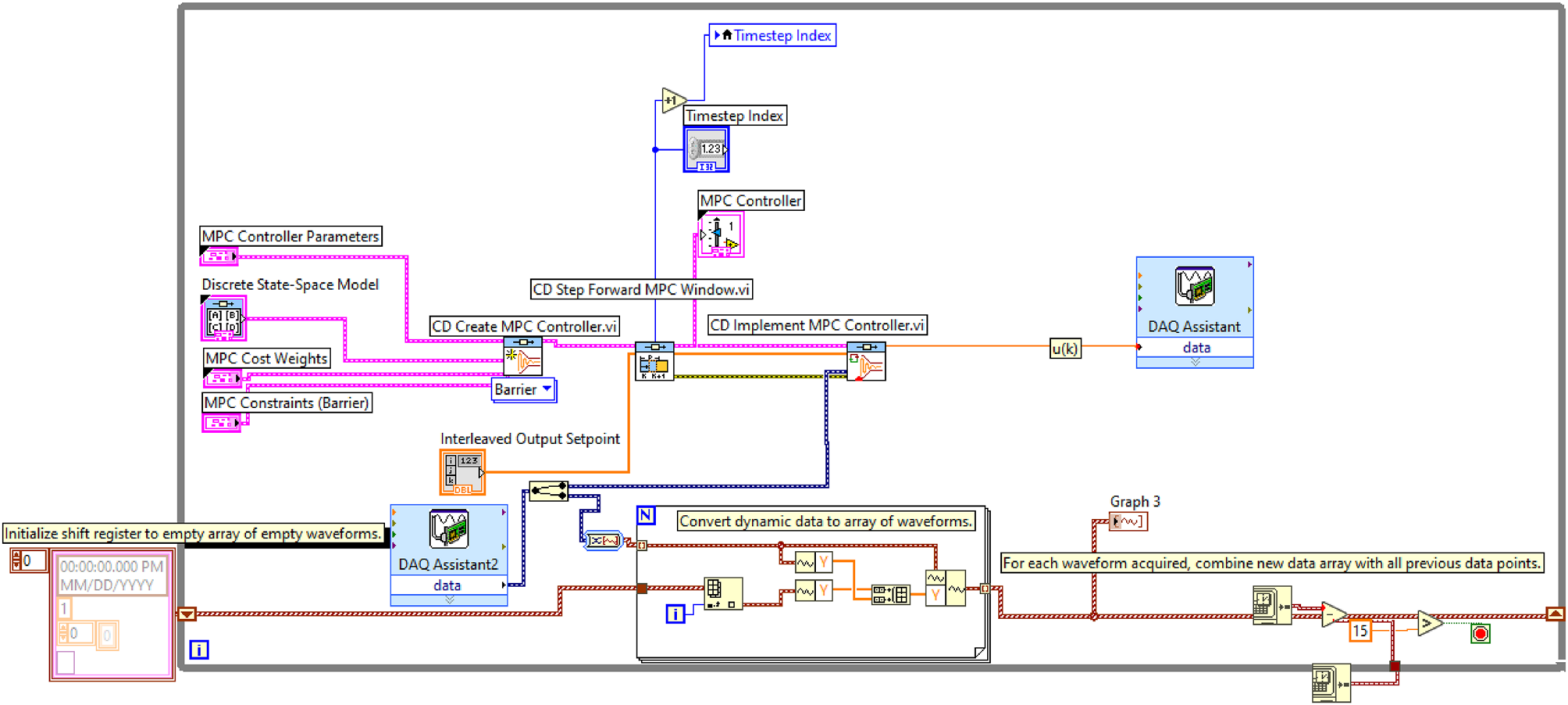
Block diagram of LabVIEW MPC.

Moving on to the practical implementation in LabVIEW, The visual representation in Figure 4 encapsulates the LabVIEW Model Predictive Control (MPC) block diagram, showcasing essential components for implementing the MPC algorithm. This depiction includes various tabs and functionalities integral to the MPC controller’s operation:

- **MPC Controller Parameters Tab:** This tab houses text boxes for inputting values crucial to the MPC algorithm, including the prediction horizon, control horizon, and initial window settings.
- **Discrete State Space Model Tab:** Within this tab, a data entry window enables the specification of the discrete state space model.
- **MPC Cost and MPC Constraints Tabs:** These tabs contain windows for defining constraints and weight factors pertinent to the MPC scheme. They facilitate the specification of limitations on input and output variables alongside cost weights, crucial for optimal control strategy determination.
- **CD Create MPC Controller VI:** This VI is utilized to generate the MPC controller. It takes as input the discrete state space model data, MPC parameters (including prediction horizon and control horizon), cost weights, and constraints. With these inputs, it creates the MPC controller.
- **CD Step Forward MPC Window VI:** This VI calculates the appropriate portion of the set point and disturbance profile while moving the control and prediction horizon forward. These windows are then connected to the CD Implement MPC Controller VI.
- **CD Implement MPC Controller VI:** This VI is responsible for calculating the control action to be applied to the system. It leverages the output reference window, the MPC controller created by the CD Create MPC Controller VI, and the measured output to compute the control action along the control horizon at time ‘k’.

The implementation of control actions is facilitated by the DAQ Assistant, enabling the execution of control signals to the system. Simultaneously, the DAQ Assistant 2 is utilized for acquiring data from the system, forming an integral part of the closed-loop control setup. Additionally, Tab control graphs are created to visually present the results obtained from the DAQ Assistant’s execution.

These LabVIEW VIs can be understood as operating in two stages. The first stage involves collecting all MPC parameters from the user, preparing the system for MPC. The second stage enforces the control actions, generated by the MPC scheme, on the plant.

### Neural network model predictive control

The neural network model predictive controller is an advanced control strategy that utilizes a neural network model of a nonlinear plant to predict its future performance. By optimizing the control input over a specified prediction horizon, the controller seeks to achieve optimal plant performance. These predictions are then employed by an optimizer to compute the control signal that minimizes the MPC’s objective function over a specified time horizon. The selection of the cost horizon value is a pivotal aspect of this control strategy and is assessed across a range from 5 to 30 to determine the optimal value that yields the most favorable results^22,23^.

**Figure 5:**
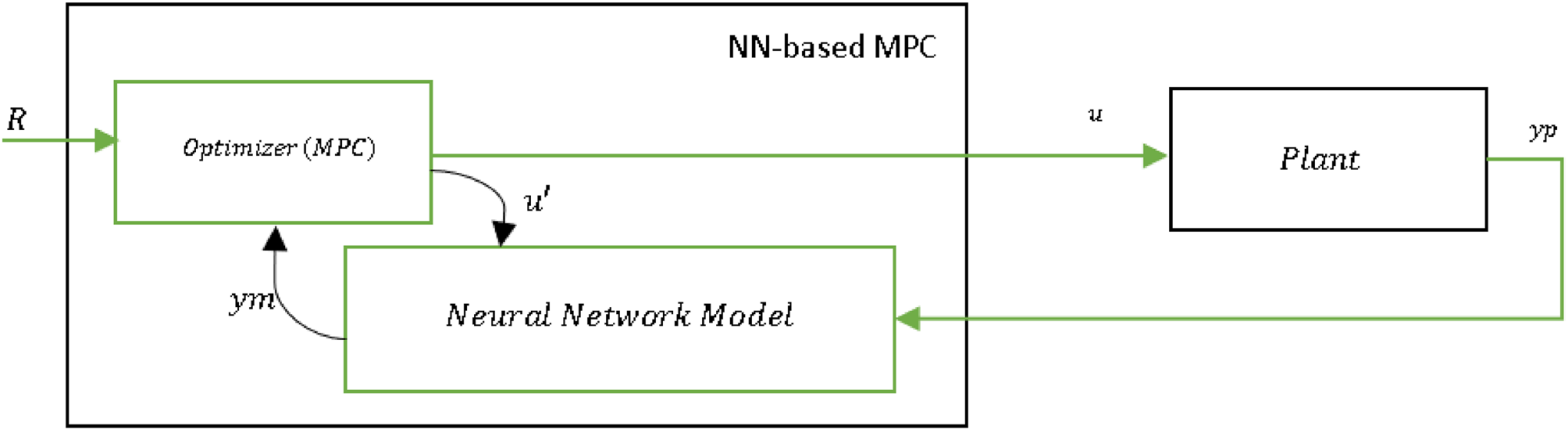
NN predictive controller block diagram.

**Figure 6:**
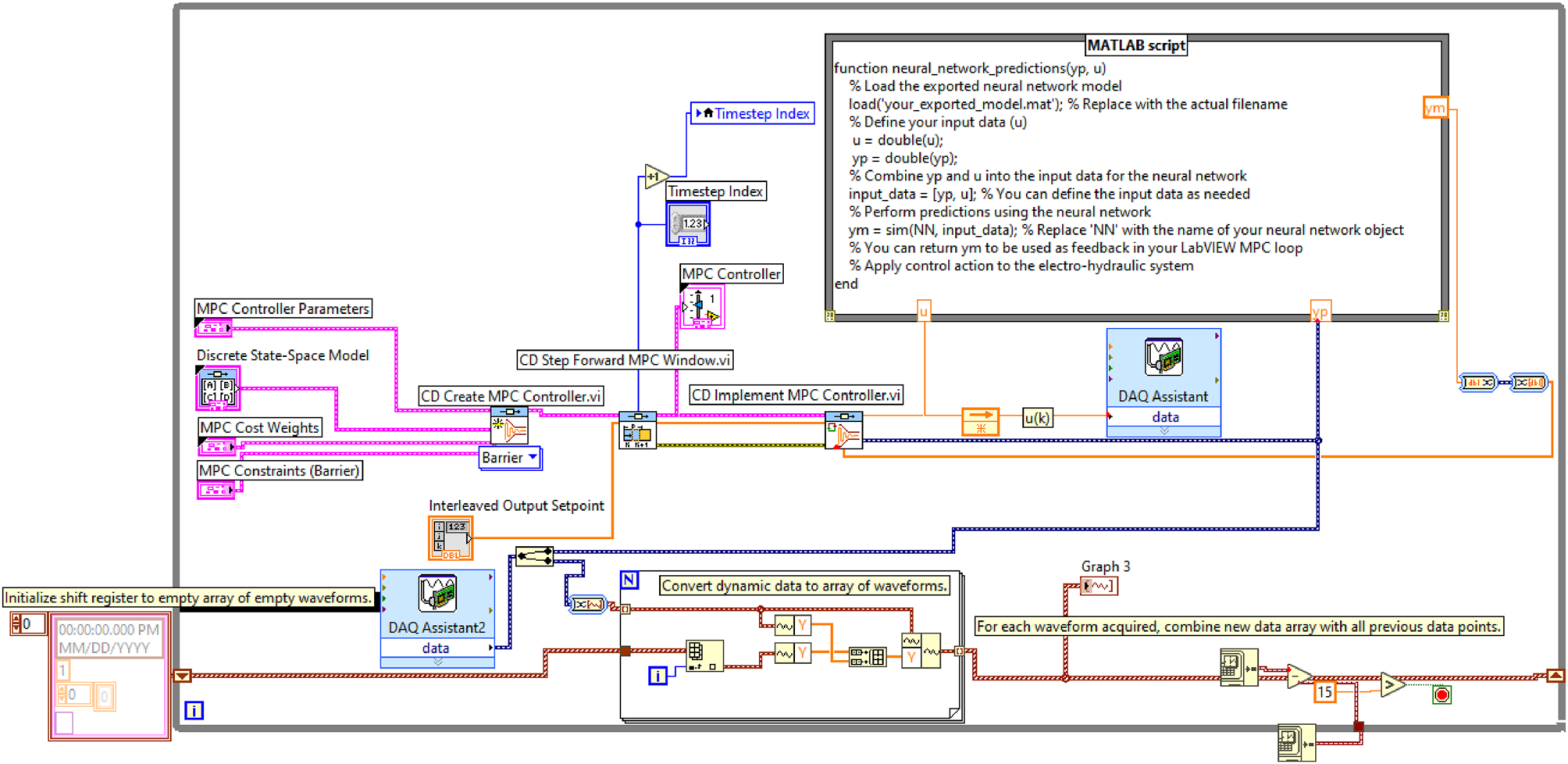
Block diagram of LabVIEW NN-MPC.

The process of model predictive control (MPC) is visualized in the block diagram shown in Fig. 5. The controller comprises two fundamental components: the neural network plant model and the optimization block. The optimization block’s primary function is to calculate the values of u’ that minimize the cost function J. Once these optimal values are determined, they are used as inputs to the plant. The neural network plant model is trained using MATLAB, and the complete controller block is implemented using LabVIEW, as depicted in Fig. 6 which illustrates the LabVIEW implementation of the Neural Network Model Predictive Control (NN-MPC), incorporating fundamental components akin to the MPC methodology described in Fig. 4. However, it incorporates an additional element—a MATLAB script serving a pivotal role in integrating the neural network model into the control architecture.

This MATLAB script’s purpose is to load the neural network model, define and combine the inputs (control action and system output), and subsequently generate predictions using the neural network. The output from the neural network, denoted as ‘ym’, can be used as feedback within the LabVIEW MPC loop, facilitating informed control decisions.

To successfully implement the NN-MPC method, a well-structured sequence of steps must be meticulously followed. The process commences by defining the system’s dynamics, which involves developing a comprehensive mathematical model that encompasses the system’s inputs, outputs, and states. Subsequently, the collection of training data begins with the aim of faithfully representing the system’s behavior. This data is pivotal for training a neural network capable of approximating the system model.

The third phase revolves around the training of the neural network using the collected dataset, enabling the network to predict the system’s output based on input and state measurements. A critical step follows, where the trained neural network is converted into a MATLAB script for seamless integration into the control architecture.

Central to the NN-MPC methodology is the design of the Model Predictive Controller (MPC). During this phase, careful consideration is given to defining the cost horizon and control horizon. This involves a delicate balance between optimizing control effort and achieving precise control objectives. Building upon the foundation laid in the previous step, constraints are established to ensure that control actions adhere to the operational limits of the system.

Before transitioning to the real-world environment, a MATLAB script representing the trained neural network is generated.

The final step involves the seamless integration of the MATLAB script into LabVIEW, facilitating the implementation of the control strategy within the electro-hydraulic system. This structured approach ensures a systematic and efficient implementation of the NN-MPC method, guaranteeing its effectiveness in controlling complex systems.

**Figure 7:**
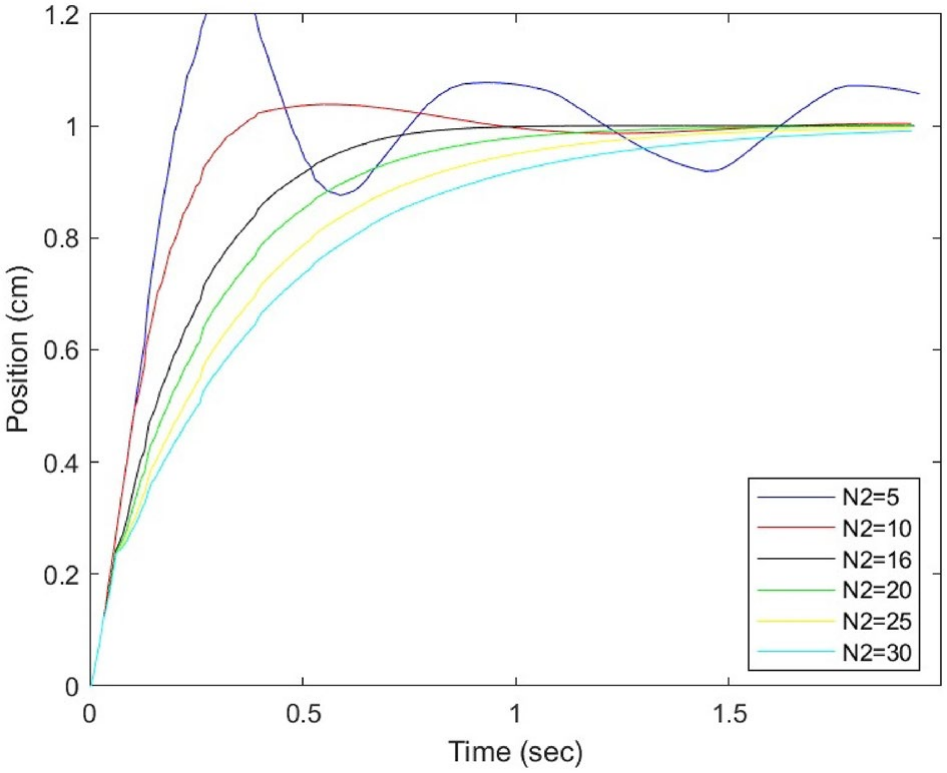
Simulation results of the NN-MPC controller by tuning prediction horizons.

Figure 7 presents the simulation results of the system using the NN-MPC control strategy, with a specific focus on tracking control performance with unit step inputs. The simulation results demonstrate that a prediction horizon of 16 yields the best performance in terms of both tracking accuracy and settling time.

Table 4 provides the parameters of the implemented neural network architecture and the MPC controller for the NN-MPC control strategy. The neural network architecture comprises three hidden layers and the training data consists of 5000 samples, with maximum and minimum input/output values defined. The training parameters include the number of epochs set to 500.

**Table 4:** Neural network MPC controller parameters.

MPC parameters			
Cost horizon	16	Control weighting factor	0.05
Control horizon	2	Iterations per sample time	2
Neural network architecture			
Hidden layer size	3	No. delayed inputs	1
Sampling interval	0.19	No. delayed outputs	1
Training data parameters			
Training samples		5000	
Maximum input	10	Maximum output	11
Minimum input	− 10	Minimum output	0.5
Maximum interval value	1	Minimum interval value	0.5
Training parameters			
Epochs		500	

## PID controller design

In the industry, PID controllers are widely utilized due to their ability to provide a performance “baseline” that can be quickly established using the Ziegler-Nichols closed-loop tuning method. The input to a plant controlled by a PID controller is calculated as shown in Eq. (20), by combining the proportional, integral, and derivative control modes. The proportional mode calculates the output based on the difference between the setpoint and the process variable, while the integral mode accumulates the error over time to reduce steady-state errors. The derivative mode calculates the output based on the rate of change of the error to improve the response time of the controller. Despite their popularity, PID controllers have some limitations and may not be the optimal choice for all applications. In such cases, more advanced control techniques such as MPC and neural network MPC may be more appropriate. These methods can provide superior control performance, but require more advanced tuning and implementation techniques.

20 $$u\left(t\right)=Kp(e dt+\frac{1}{Ti}\int e dt+Td\frac{de}{dt})$$

This method involves calculating the ultimate gain value, Kcr, and the ultimate period of oscillation, Pcr, which are then used to derive the PID parameters. In our system, we obtain the transfer function and use the ss2tf function in Matlab to apply the closed loop in Simulink and determine the values of Kcr and Pcr. Specifically, we found that Kcr was 8.3 and Pcr was 256s. To calculate the PID parameters, we followed the equations from (21) to (23) derived from the Ziegler-Nichols method^24,25^.

21 $$Kp=0.6 Kcr=5$$

22 $$Ti=0.5 Pcr=128$$

23 $$Ti=0.125 Pcr=32$$

### Simulation

**Figure 8:**
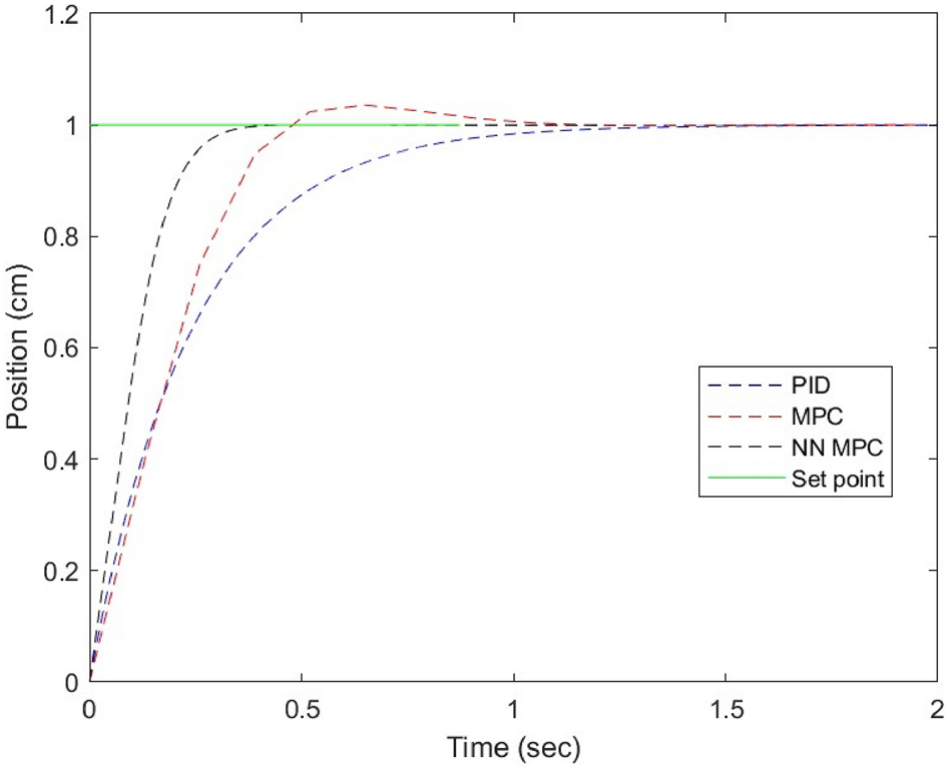
Simulation results of the NN-MPC controller by tuning prediction horizons.

In order to optimize controller algorithms and determine the most effective parameters, MATLAB Simulink is employed. Through simulations, it was observed that NN MPC achieved stability in the shortest amount of time, with zero overshoot, after just 0.387 seconds. This was followed by MPC, which exhibited a slight overshoot of 3.58% after 1.19 seconds. On the other hand, the PID controller required the longest amount of time to stabilize, doing so after 1.39 seconds. The step response of each controller can be visualized in Figure 8.

## The experiment and result

The upcoming section of our research paper will focus on two primary aspects related to the performance analysis of hydraulic circuits. The section will be divided into two parts, with the first part focusing on a comparison between different control techniques under varying loads. This comparative analysis aims to highlight the efficiency and effectiveness of different control techniques and their impact on the overall performance of hydraulic circuits. The second part of this section will focus on an energy consumption comparison between different hydraulic circuits. Our research findings provide a valuable understanding of the energy consumption of hydraulic circuits utilizing various control techniques. This comparison aims to showcase the potential benefits of different control techniques in enhancing energy efficiency, leading to more sustainable and cost-effective energy consumption.

### The results of the control techniques under different load

The LabVIEW software was employed to apply PID, MPC, and NNPC control techniques to the system. The performance of the PID and MPC controllers was compared to that of the designed NN MPC. To evaluate the system’s response, step input displacements with an amplitude of 100 mm were applied under different loads, including no-load and 7.5 MPa. The effectiveness of each controller was assessed by analyzing their ability to regulate the system output in response to the input changes. The results of these tests were analyzed to determine which controller was most effective in achieving the desired control objective.

### Noload input

**Figure 9:**
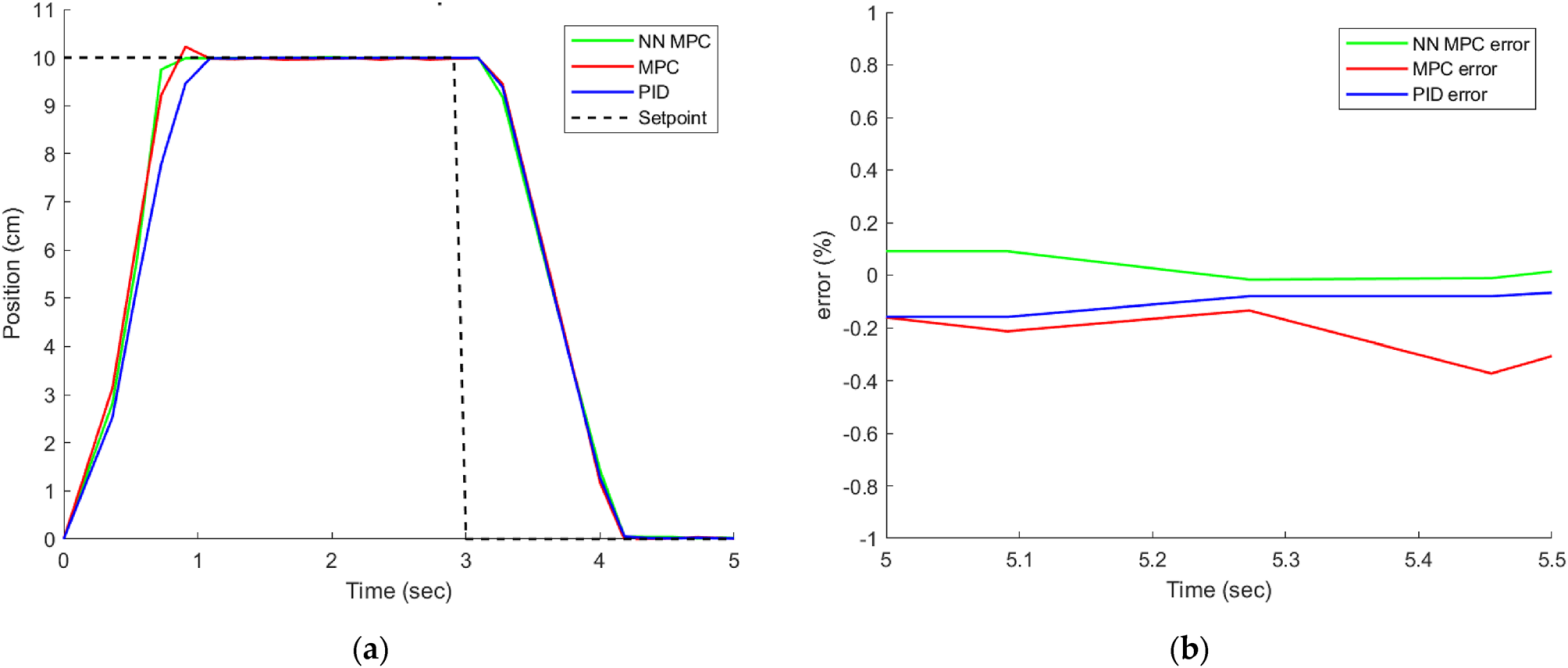
(**a**) System response at No-load. (**b**) Position error under No-load.

Figure 9 displays the response and trajectory error of the system when subjected to a 100 mm reference input. It can be observed that the NN MPC outperforms both the PID and MPC controllers in terms of settling time. Moreover, the NN MPC exhibits a higher level of accuracy in tracking the setpoint as compared to the other controllers, which exhibit slight overshoot. Therefore, it can be concluded that the NN MPC is the most effective controller for the given system.

### 7.5 MPa load input

**Figure 10:**
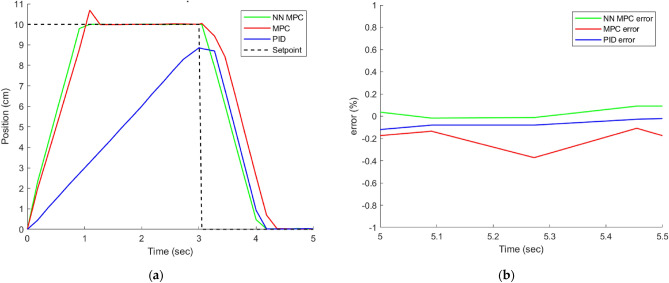
(**a**) System response under load 7.5 MPa. (**b**) Position error under load 7.5 MPa.

**Figure 11:**
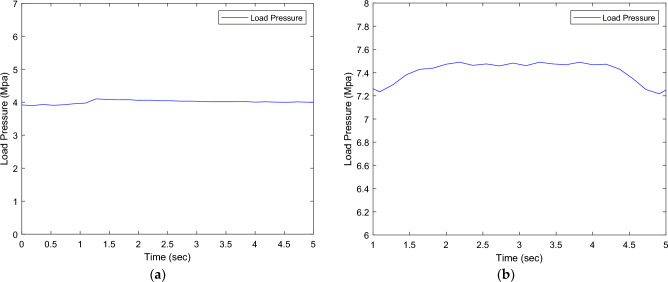
Pressure sensor reading of different loads.

Upon observing the performance of the system while a constant 7.5 MPa load is applied, as depicted in Figure 11, the system’s operation and tracking error are analyzed. Figure 10 displays the system’s output response to the input, and it is evident that the NN MPC controller outperforms both the MPC and PID controllers in terms of tracking error and settling. The PID controller, on the other hand, fails to achieve the desired displacement of 100 mm and retracts before reaching it.

Figures 11 illustrates the pressure sensor measurements for different magnitudes of loads.

The experimental results are displayed in Table 5.

**Table 5:** Comparison between controllers under different loads.

Control	Load (MPa)	Settling time (sec.)	Overshoot %	Error %
PID	–	1.09	0	− 0.159
MPC		1.09	2.037	− 0.213
NN MPC		0.909	0	− 0.011
[9]		1.7	0	−
[10]		–	–	0.5201
PID	7.5	3.63	0	− 1.98
MPC		1.45	6.27	0.26
NN MPC		1.09	0	− 0.012

### Power consumption comparison

The amount of power consumed by the hydraulic system can be mathematically represented by Eq. (24).

24 $$P={\int }_{t}^{t+\Delta t}{\text{pQdt}}$$

where, $P$ is Power consumed by the hydraulic system, $p$ is the measurement of pressure sensor (4), $Q$ is the measurement of flow sensor (5), $t$ is the starting time of the experiment, $\Delta t$ is time duration of the experiment cycle.

**Figure 12:**
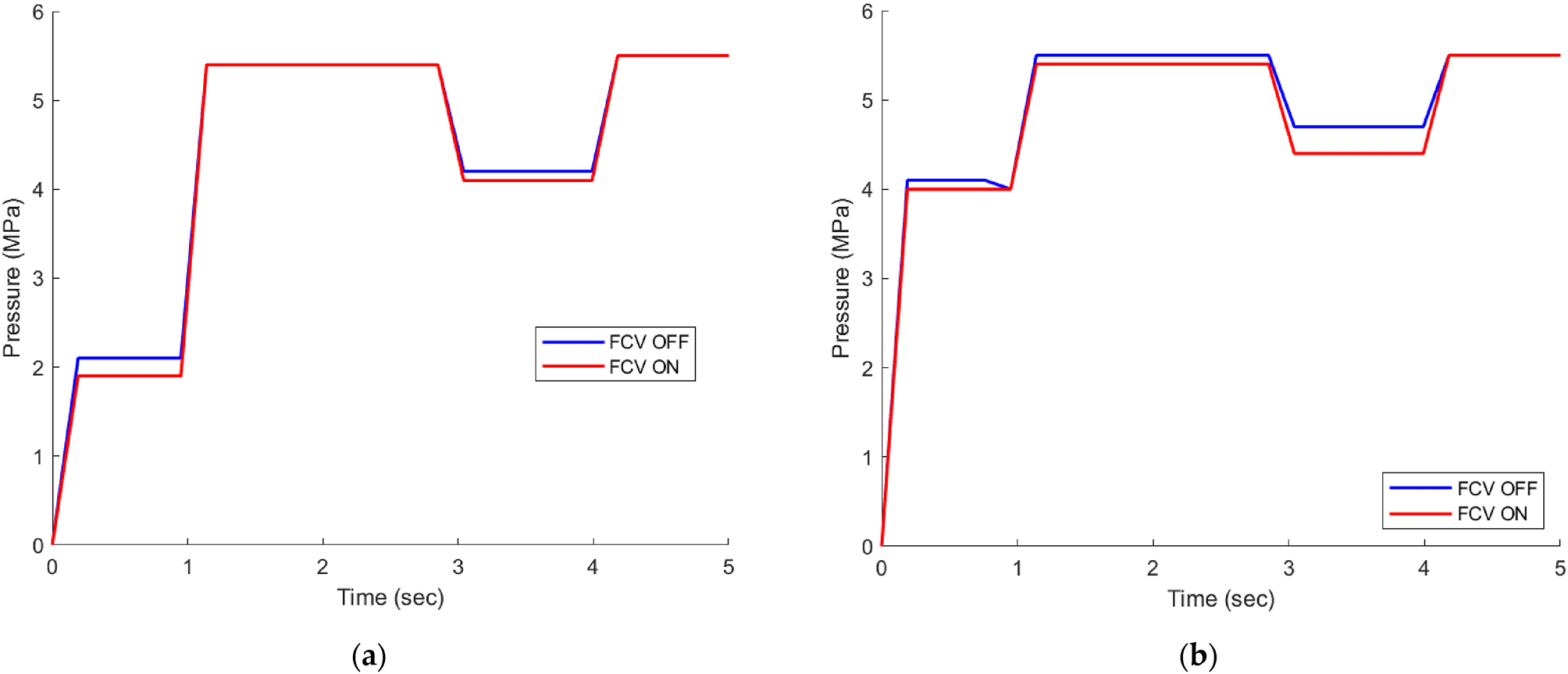
(**a**) Pressure measured by pressure sensor at No-load. (**b**) Pressure measured by pressure sensor at 7.5 MPA.

**Figure 13:**
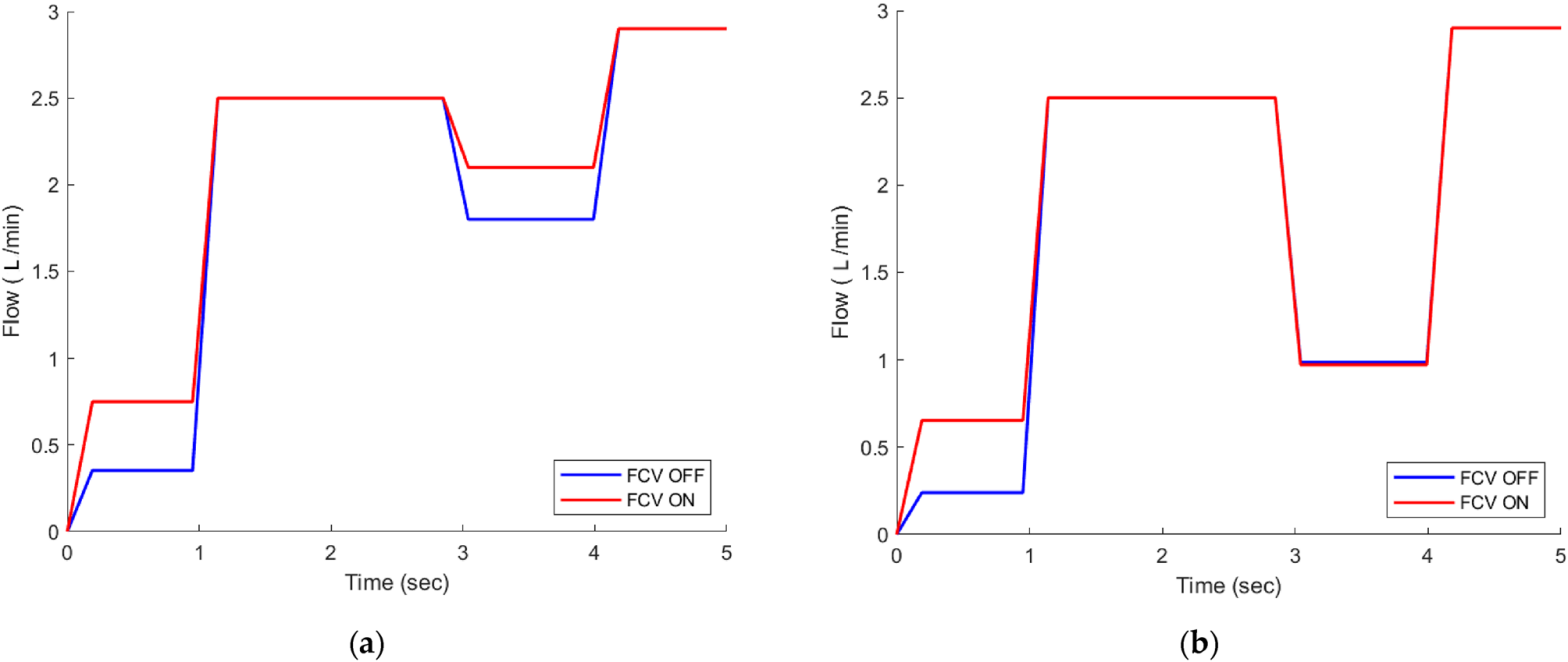
(**a**) Flow rate through PRV at No-load. (**b**) Flow rate through PRV at 7.5 MPa.

**Figure 14:**
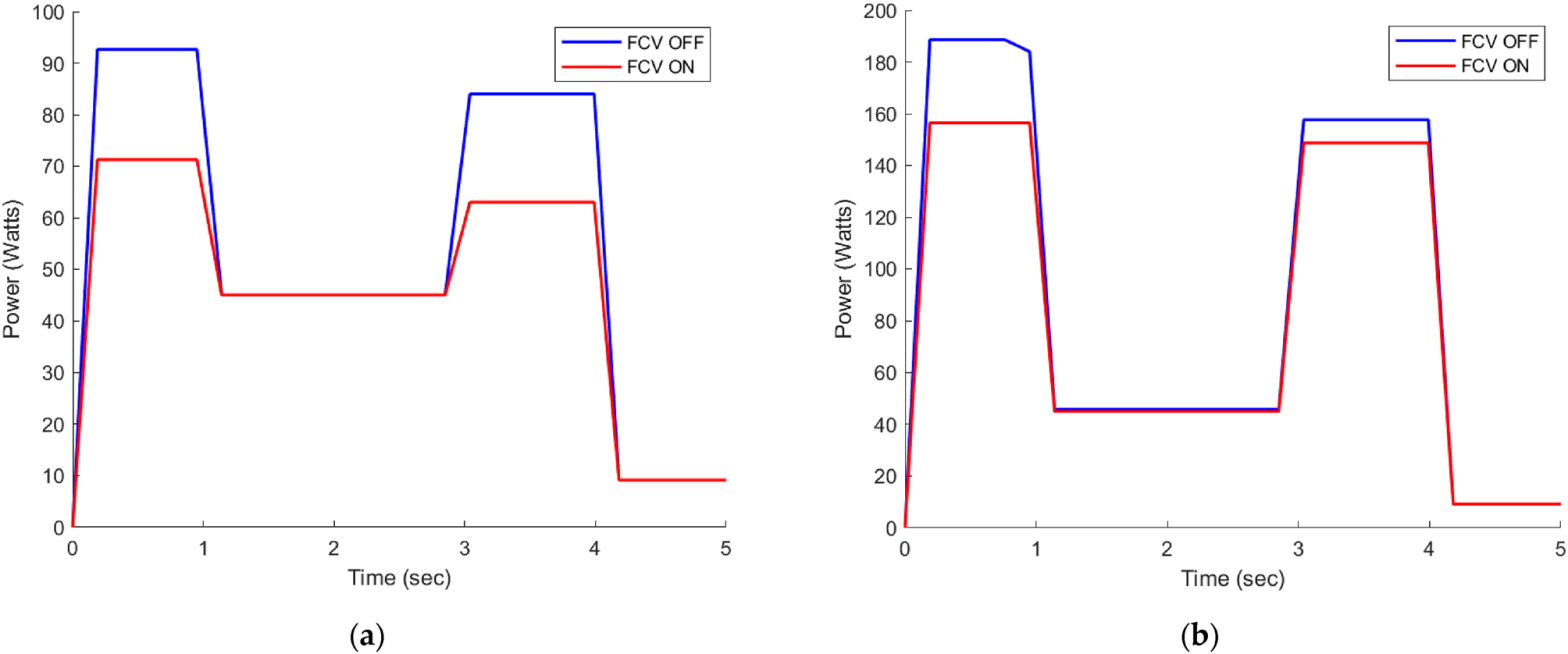
(**a**) Power consumption at No-load. (b) Power consumption at 7.5 MPa.

In the context of position control of a cylinder using a flow control valve, Fig. 13 depicts the flow behavior through the pressure relief valve (PRV) (8). At the beginning of the process, the cylinder is stationary, and its movement is monitored while the pressure sensor (4) records the PRV’s cracking pressure (50 bar) shown in Fig. 12. Additionally, the flow sensor (5) measures the flow rate. As the cylinder advances and then returns to its original position, both the pressure and flow show fluctuations (Figs. 13 and 14).

Figure 14 clearly shows that the circuit utilizing FCV technology exhibits significantly lower energy consumption in comparison to the circuit without FCV. The observed reduction in energy consumption can be attributed to the discharge of excess flow in the circuit using PDCV, which occurs through the pressure relief valve at its cracking pressure, as illustrated in Fig. 12. However, with the use of FCV technology, the excess flow is expertly bypassed at the load pressure, resulting in a remarkable increase in efficiency of about 15.35% at No-load and 9% at load 7.5 MPa, as described in detail in Table 6. It is worth noting that the term FCV ON refers to the fully open state of the FCV, while FCV OFF indicates that the FCV is closed.

**Table 6:** Average energy consumption under different loads.

System	Energy consumption (J) at No-load	Energy consumption (J) at load 7.5 MPa
System without using FCV	235.87	380.22
Proposed system using FCV	199.66	345.85
Energy saving	15.35%	9%

Our findings underscore the enormous potential of FCV technology in improving the energy efficiency of hydraulic systems, leading to a more sustainable and cost-effective approach to energy consumption. These results have significant implications for various industries and pave the way for the widespread adoption of FCV technology.

**Figure 15:**
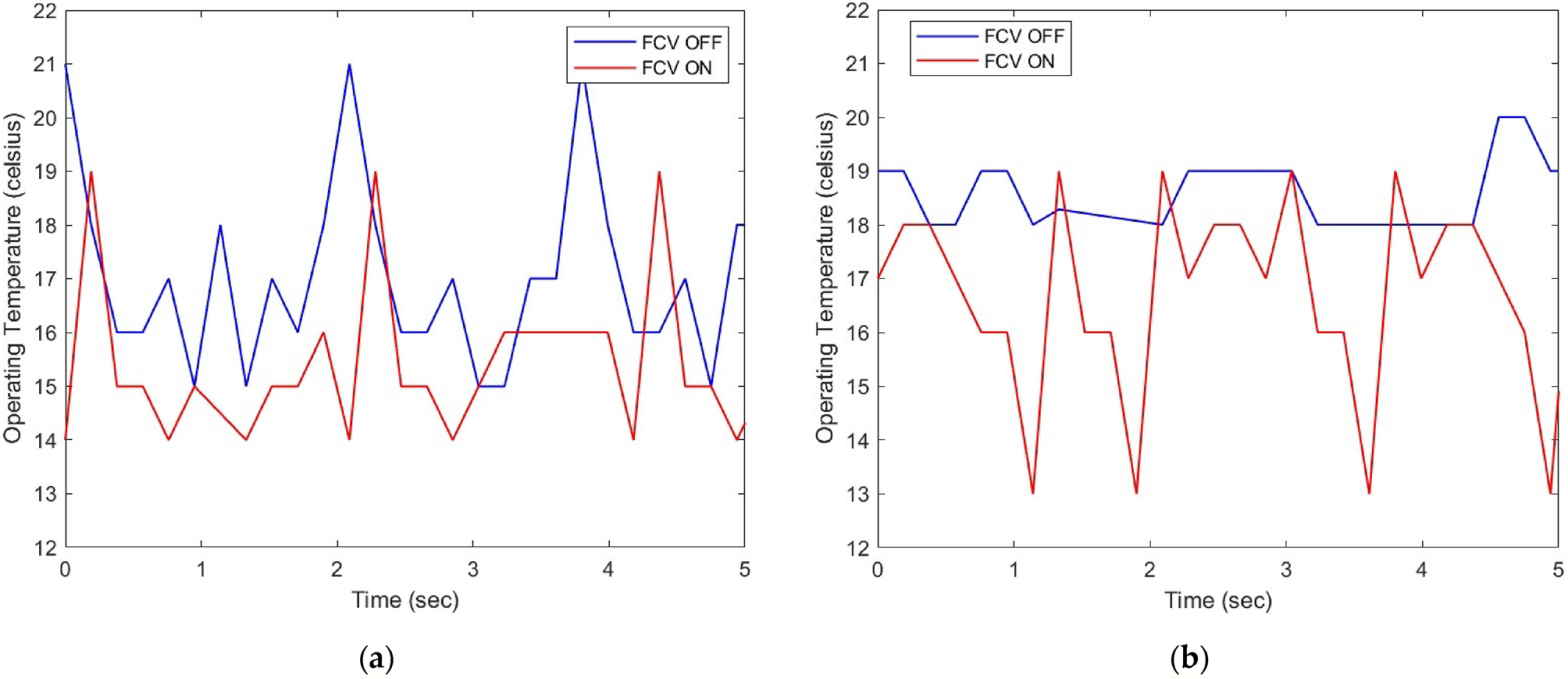
(**a**) Operating temperature at No-load. (**b**) Operating temperature at 7.5 MPa.

To ensure the reliability and accuracy of our findings, further verification was carried out through the use of advanced temperature sensors to measure the operating temperature of the system. As shown in Fig. 15, our results indicate a significant decrease in the average operating temperature of the system by 10% when utilizing FCV technology. This reduction in temperature not only highlights the superior efficiency and performance of FCVs but also showcases their potential for widespread implementation in a variety of settings. Our findings provide compelling evidence of the benefits of FCVs and their ability to enhance the overall sustainability and effectiveness of energy systems.

## Conclusion

This research investigates methods to enhance hydraulic system power consumption and performance through advanced control techniques and technology. The comparison between PID, MPC, and NN MPC controllers revealed NN MPC’s superiority in speed, accuracy, and minimizing overshooting across various loads, suggesting its potential to surpass traditional controller limitations. This precision and stability are crucial for industrial applications relying on hydraulic systems. Additionally, exploring energy consumption, particularly by integrating Flow Control Valve (FCV) as a hydraulic bypass, demonstrates substantial potential for energy savings across diverse hydraulic applications, from heavy machinery to manufacturing and construction. Circuits incorporating FCVs exhibited impressive energy savings of approximately 15.35% with no load and 9% under a 7.5 MPa load compared to those without FCV. Moreover, the use of FCV was associated with a significant 10% reduction in operating temperatures, underscoring the efficiency and sustainability benefits these technologies offer hydraulic systems.

### Ethics approval and consent to participate

This research, which pertains to the optimization of power consumption in electrohydraulic position systems, did not involve human or animal subjects. As such, formal ethics approval and informed consent were not applicable to this study. The investigation primarily concentrated on the technical aspects and performance enhancements of electrohydraulic systems.

## Data Availability

We acknowledge the requirement of a data availability statement for this journal. The data used in our study, including sensor calibration data, LabVIEW files and experimental results, can be obtained from the repository mentioned in the article. Researchers can access and analyze this data to validate and build upon our findings. The data and material associated with our research are available in the repository mentioned in the article. Researchers can access and download the code, data, and resources from the following link: https://github.com/Omar-H-Khedr/Advanced-Control-Systems-for-Enhanced-Motion-Tracking-in-Electro-Hydraulic-Systems.git.

## Code availability

We have ensured the availability of the code related to our research. Researchers can find the code in the repository mentioned above. It includes LabVIEW files, MATLAB files, and the Position Figures Generator code, which facilitate the implementation, analysis, and visualization of the control systems for electro-hydraulic systems. Declarations Conflict of interest the authors declare no conflict of interest.
